# Noble metal catalyst detection in rocks using machine-learning: The future to low-cost, green energy materials?

**DOI:** 10.1038/s41598-023-30822-6

**Published:** 2023-03-07

**Authors:** Elena Ifandi, Daphne Teck Ching Lai, Stavros Kalaitzidis, Muhammad Saifullah Abu Bakar, Tassos Grammatikopoulos, Chun-Kit Lai, Basilios Tsikouras

**Affiliations:** 1 Geosciences Programme, Faculty of Science, Universiti Brunei Darussalam, Gadong, BE1410 Brunei Darussalam; 2 School of Digital Science, Universiti Brunei Darussalam, Gadong, BE1410 Brunei Darussalam; 3 Department of Geology, University of Patras, 26504 Rio-Patras, Greece; 4 Faculty of Integrated Technologies, Universiti Brunei Darussalam, Jalan Tungku Link, BE1410 Brunei Darussalam; 5 SGS Canada Inc., 185 Concession Street, Lakefield, ON K0L 2H0 Canada; 6 Fortescue Metals Group Ltd., 87 Adelaide Terrace, East Perth, WA 6004 Australia

**Keywords:** Heterogeneous catalysis, Geochemistry, Computational science

## Abstract

Carbon capture and catalytic conversion to methane is promising for carbon–neutral energy production. Precious metals catalysts are highly efficient; yet they have several significant drawbacks including high cost, scarcity, environmental impact from the mining and intense processing requirements. Previous experimental studies and the current analytical work show that refractory grade chromitites (chromium rich rocks with Al_2_O_3_ > 20% and Cr_2_O_3_ + Al_2_O_3_ > 60%) with certain noble metal concentrations (i.e., Ir: 17–45 ppb, Ru: 73–178 ppb) catalyse Sabatier reactions and produce abiotic methane; a process which has not been investigated at the industrial scale. Thus, a natural source (chromitites) hosting noble metals might be used instead of concentrating noble metals for catalysis. Stochastic machine-learning algorithms show that among the various phases, the noble metal alloys are natural methanation catalysts. Such alloys form when pre-existing platinum group minerals (PGM) are chemically destructed. Chemical destruction of existing PGM results to mass loss forming locally a nano-porous surface. The chromium-rich spinel phases, hosting the PGM inclusions, are subsequently a second-tier support. The current work is the first multi-disciplinary research showing that noble metal alloys within chromium-rich rocks are double-supported, Sabatier catalysts. Thus, such sources could be a promising material in the search of low-cost, sustainable materials for green energy production.

## Introduction

The Paris Agreement highlights the paramount importance of establishing sustainable fuel sources. Catalytic hydrogenation of carbon dioxide is a promising carbon–neutral fuel source^1^. Emerging research on sustainable energy and environment protection, and implementation of green policies from governments and international foundations^2–4^ emphasize the need to shift towards environmentally friendly energy production.

The Sabatier reaction (Eq. 1) is a well-known and widely used process to produce methane from catalytic hydrogenation of CO_2_. It is a two-step reaction, involving the combination of an endothermic reversed water gas shift (RWGS) reaction and an exothermic CO hydrogenation (Eqs. 2 and 3, respectively), at elevated pressures and temperatures ranging between 200 and 500 °C^5^.

1 $${\text{CO}}_{{2}} + {\text{ 4H}}_{{2}} \to {\text{ CH}}_{{4}} + {\text{2H}}_{{2}} {\text{O}}; \, \Delta {\text{H }} = - {165 }\;{\text{kJ}}/{\text{mol}}$$

2 $${\text{H}}_{{2}} + {\text{ CO}}_{{2}} \to {\text{ CO}} + {\text{H}}_{{2}} {\text{O}}; \, \Delta {\text{H }} = { 41}\;{\text{ kJ}}/{\text{mol}}$$

3 $${\text{3H}}_{{2}} + {\text{ CO }} \to {\text{ CH}}_{{4}} + {\text{ H}}_{{2}} {\text{O}}; \, \Delta {\text{H }} = - {2}0{6}\;{\text{kJ}}/{\text{mol}}$$

The produced hydrocarbon is not exclusively methane but a mixture of hydrocarbons and other organic molecules depending on the activity and selectivity of the catalyst. Nickel and ruthenium-based catalysts produce almost exclusively methane. Less reactive metal catalysts (Pd, Pt, Rh, Mo, Re, Au) produce simultaneously CH_4_, CH_3_OH and CO via the RWGS^5^. In previous studies, the lowest reported temperature for CO_2_ hydrogenation was at room temperature (25 °C). A ruthenium nanoparticle loaded on a TiO_2_ catalyst, lead to methane formation within the first 5 min of the experiment^6^.

Interestingly, low temperature (< 100 °C) CO_2_ hydrogenation occurs in nature, producing abiotic methane (methane hereafter) via Sabatier reaction. Studies suggest that the source of methane is chromium-rich rocks (chromitites)^7,8^. Minerals with catalytic properties within chromitites are particularly promising in producing commercially efficient and sustainable catalysts. Mineral catalysts could reduce the cost and environmental impact related to the synthesis of catalysts (e.g., less processing) and fuel production (lower energy for lower-temperature reactions). There is currently limited understanding on the constrains during low temperature methane formation. Direct evidence for the kinetics of methane formation in nature is limited. The existing studies on high-temperature (> 300 °C) experiments are not representative of the methanation in chromitites. Isotopic analyses of methane in ruthenium-bearing chromitites suggest that methane was formed below 150 °C^7–9^. Low temperature (< 100 °C) experiments demonstrated that pure ruthenium catalysts, in quantities equivalent to their natural occurrence in chromitites, effectively support methanation^10^. Hence the original hypothesis was that the most abundant ruthenium phase in chromitites should be the catalyst. Ruthenium-rich phases occur mainly in chromitites including laurite (RuS_2_), laurite-erlichmanite (OsS_2_) solid solutions, and Ir–Ru–Os–Ni alloys (IPGE-Ni alloys)^11–13^. However, the exact *locus* of methane generation and the actual catalyst(s) is poorly understood.

Natural materials including chromitites have a complex chemical history. Over the span of millions of years, rocks undergo numerous chemical transformations altering their original chemical composition along with their constituent minerals. Methane formation in chromitites comprises a minor part of the overall rock evolution. In a mathematical context, chromitites are multivariate systems. The abundance and distribution of the measured variables (i.e., chemical elements) derive from multiple overlapping processes. Variables are inheritably interrelated, having contrastingly different variances and scales. In this context, variables related to methane formation have low variance. Thus, it is critical to apply a suitable data analysis method to extract information on the mineral catalyst.

The workflow of this study is sequential: large-scale target – source rock modelling – catalyst prediction – micro-scale target. The rocks with high potential (chromitites) are derived from abandoned chromium ore mines in Greece. Large-scale inferences are based on whole-rock chemical analyses. However, chemical data from rock samples are compositional (i.e., closure to 100%) and thus, linear regression and other parametric methods are unsuitable to detect causal effects. The suitability of non-parametric and stochastic approaches was tested using ANOVA and Spearman's correlations on the whole-rock chemical analyses to compare to methane concentrations in various rock-types and indicated the methane source rock.

A combination of stochastic machine-learning algorithms including Random Forest Regression (RFR), t-distribution Stochastic Neighbour Embedding (t-SNE), and model-based clustering with Bayesian Information Criterion (mBIC), determined the element proxies of the mineral catalyst. The RFR classified the variables related to methane in order of importance. Reverse RFR modelling attested the predictors in identifying rocks with high methane levels. The best predictors comprise the potential catalyst proxies. Additional machine-learning algorithms (t-SNE and mBIC) verify the robustness of the identified catalyst proxies. The micro-scale investigation focuses in the richest-in-methane chromitites. A series of quantitative mineralogical analyses and observations (i.e., composition, stoichiometry, reaction indicators, chemical or crystal lattice modifications) connects the identified catalyst proxy to specific minerals. The latter comprise the suggested low-cost catalysts.

Stochastic machine-learning techniques were used to trace the naturally occurring catalysts among the 55 analysed chemical elements. Herein, we suggest a novel, natural catalyst, in line with the need for a cost-effective catalyst that is active under low temperatures, resulting to lower environmental impact for its processing. The concept of a double-supported catalyst is also introduced. The present work is the first multi-disciplinary approach showing that noble metals alloys (i.e., Ir, Ru) within chromium-rich rocks are double-supported, Sabatier catalysts.

## Results

### Catalyst host rock

The analysed samples are grouped as basic (52–45 wt.% SiO_2_), ultrabasic (SiO_2_ < 45 wt.%) and chromitites (ore deposits deficient in SiO_2_ and with high amounts of Cr_2_O_3_ = 30–70 wt.%). Subgroups within the basic lithotypes are the basic volcanic rocks and rodingitised basic rocks. The progressive decrease of SiO_2_ and increase in Cr_2_O_3_ are coupled with enrichments in MgO, and FeO (Supplementary Tables 1 and 2). ANOVA tests between the lithotypes and methane contents showed that the rock type has a large effect on the methane concentration (F (4, 53) = 53.935, *p* < 0.001, ω^2^ = 0.785, Supplementary Table 3). Chromitites, which host the highest amounts of methane, differ significantly from all other lithotypes with lower methane concentrations. The richer-in-methane chromitites have Al_2_O_3_ > 20%, Cr_2_O_3_ + Al_2_O_3_ > 60% and are refractory. Spearman's correlations between methane and the chemical composition of each sample revealed significant positive correlations of methane with Cr, Fe_2_O_3_, MgO, Ru, Ir, Au, Co, MnO, Ni, Pd, Pt, Rh, V, W, Zn, elements variably present in chromitites. All the positively correlated elements are reported in the literature as constituents of Sabatier catalysts^14^. Among these elements, Ir and Ru show the highest significant correlations with methane (0.932 and 0.910, respectively, Supplementary Table 4). The samples with highest concentrations in both iridium and ruthenium (Ir: 17–45 ppb, Ru: 73–178 ppb) are refractory chromitites.

### Catalyst proxies

Minerals consist of chemical elements in ordered crystallographic arrangement, which are incorporated in their crystal lattice. Therefore, the bulk chemical composition of a rock system reflects its mineralogical composition. This interdependency among elements, minerals, and rocks results in collinearity because the variables are not independent. Common parametric statistical tools such as regression analysis, especially on untreated data, are prone to biased results^14^. Furthermore, distance-based, machine-learning techniques, assuming a Euclidean geometry, are not appropriate, as the relevant assumption criteria are not met, and require complex data transformation techniques^15^. However, data treatment results in loss of information when the information is carried by features with small variances^16^, or may result in variables that are difficult to interpret. A Random Forest Regression algorithm was used to shortlist and rank the most important features (i.e., elements) in predicting the methane concentration in the different rock types. Given the stochastic approach of this method, the RFR was repeated 30 times. A mean of 0.763 for train R^2^ and 0.618 for the test R^2^ (Supplementary Tables 5–7 is reported). The best model has a train and test R^2^ of 0.808 and 0.800, respectively. The top 14 features are Ir, Ru, Cr, SiO_2_, Zn, Ge, Al_2_O_3_, Au, Co, Pt, Os, Rh, W and Fe_2_O_3_, in decreasing importance (Supplementary Tables 8–9).

**Figure 1:**
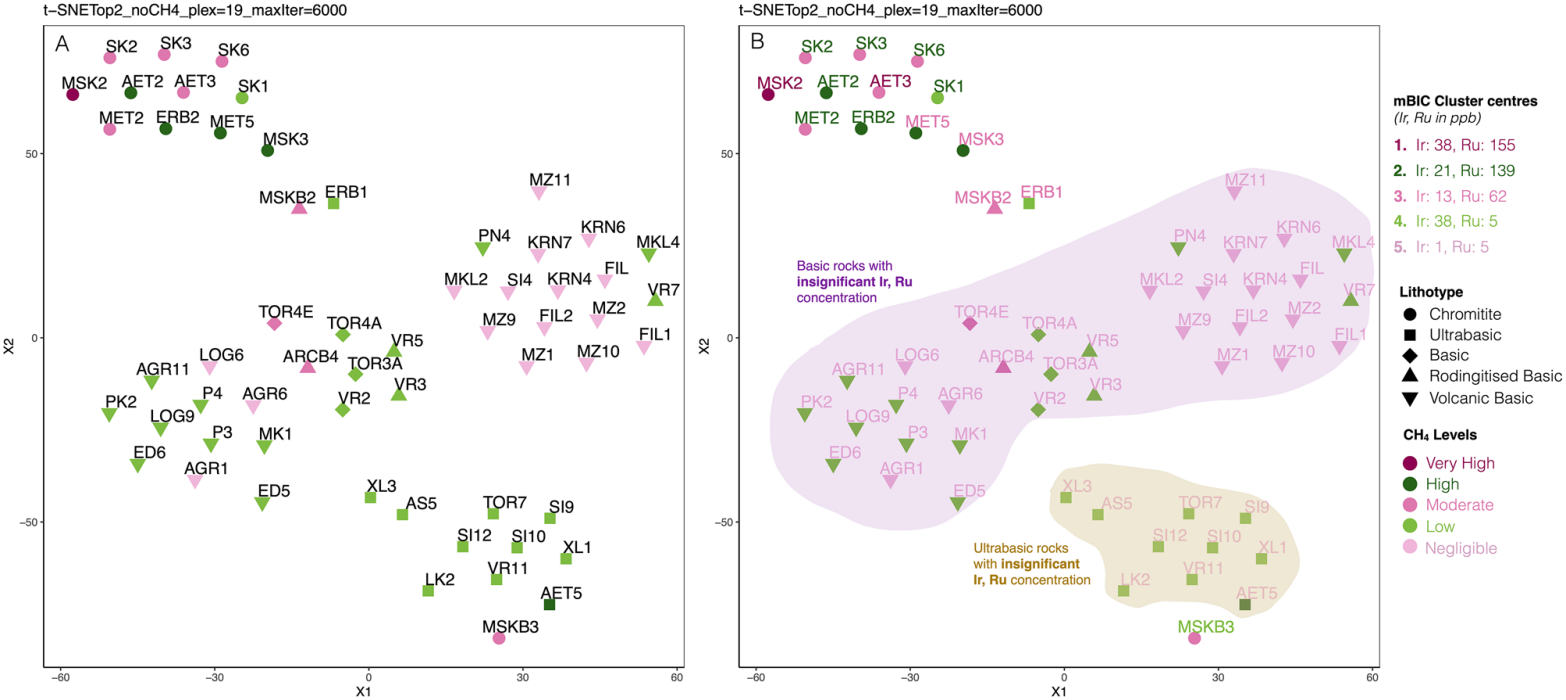
Optimal t-SNE plots of the potential catalyst proxies at 6000 iterations. (**A**) Top 2 Features (Ir, Ru) provide the ideal classification of the expected methane levels in the rock samples. (**B**) mBIC clustering over-imposed on the Top 2 features t-SNE plot. Abbreviations, plex: perplexity value, maxIter: maximum iterations (repetitions) of the algorithm.

Average rank position plots of each important feature against its sum ranking reveal distinct top feature subgroups (Fig. 1). Contrary to the expectations from previous studies^7,10^, iridium is the top predictor and more important than ruthenium in predicting methane concentration. Iridium and ruthenium comprise the most important features in methane prospecting as denoted by the steep difference in their average ranks (3.7 and 6.5, respectively vs 12.7 or higher for the rest elements) and their minimum ranking fluctuations (0 and 2.8). Hence, it appears that iridium and ruthenium are the true catalyst indicators. The rest of the elements are mostly classifiers of lithotypes with high or low methane concentrations (e.g., Cr and SiO_2_ are the proxies for chromitites and basic/ultrabasic rocks, respectively). This agrees with the well-accepted geological proxies for such lithotypes and with the current ANOVA results.

### Validation of the catalyst proxies

The hypothesis that iridium and ruthenium are the catalysts by performing sequential t-SNE plots for each subgroup of the top features (Supplementary Table 9) was investigated. The methane levels are defined according to the measured concentrations to reveal which subgroup of elements provides distinct grouping in samples with similar methane levels (Table 1). The t-SNE method was preferred because its stochastic approach allows the investigation of variables with different scales and variances. There are no assumptions about the underlying data distribution because outlier values do not affect it. Only the t-SNE that includes iridium and ruthenium (top 2 features plot) gave an almost ideal sample grouping (Fig. 1A) for most of the tested perplexity values. Methane was excluded from the t-SNE calculations, and plots give an unbiased grouping of the rocks with the highest catalytic potential.

**Table 1:** Methane concentration levels.

CH_4_ (ppmv)	Methane level
10, 000 < CH_4_	Very high
5,000 < CH_4_ ≤ 10,000	High
1,000 < CH_4_ ≤ 5000	Moderate
100 < CH_4_ ≤ 1000	Low
CH_4_ ≤ 100	Negligible

Additionally, the modified Bayesian Information Criterion (mBIC) clustering for all the subgroups of the top features (Supplementary Table 10) shows that iridium and ruthenium provide the best clustering for the methane level prediction (Fig. 1B). The clusters were compared with both the predefined methane levels and the lithotypes. Multiple association measurements (Likelihood ratio, Pearson, Contingency Coefficient, Cramer's V) for each feature subgroup (Supplementary Table 11) were used for these comparisons.

An increase of features (i.e., significant elements) in the plots results only in groups, which are interpretable from the rock-type perspective. The results strongly support the conclusion that only iridium and ruthenium are the true catalyst proxies. Although the clustering results are not as ideal as the lone t-SNE grouping, they clearly show that iridium and ruthenium concentrations in chromitites control, almost exclusively, methane abundance.

### Microscopic description of the methane source-chromitite

The chromitites with the highest concentrations of methane (Fig. 1, MSK code) were selected to assess the validity of the catalyst proxy. Detailed, microscopic characterisation revealed that Mg–Cr spinels (magnesiochromite) comprise on average 95% of the modal percentage in these rocks. The shape of the spinel crystals ranges from euhedral (hence relatively unmodified) through subhedral to anhedral grains with irregular boundaries and an average size of 200 μm. The rims of the Mg–Cr spinels are commonly altered to ferrian chromite [Fe^2+^(Cr,Fe^3+^)_2_O_4_] and magnetite (Fe^2+^${\mathrm{Fe}}_{2}^{3+}$ O_4_). Other phase transformation products (i.e., alteration) in these chromitites resulted in an overall volume decrease, leaving void spaces of up to 100 μm in the rocks. These pores are of particular interest because they are potential paths of fluid circulation and traps of abiotic gas. The voids are, less commonly, filled with newly formed phases, including chlorite [(Mg,Fe)_3_(Si,Al)_4_O_10_(OH)_2_·(Mg,Fe)_3_(OH)_6_], titanite (CaTiSiO_5_), millerite (NiS), pentlandite [(Fe,Ni)_9_S_8_], and quartz (SiO_2_).

### Characterisation of noble metal phases

Platinum-group minerals (PGM) are the main hosts of platinum-group elements (PGE: Pt, Pd, Rh, Ir, Ru, Os). Therefore, PGM heavy-mineral concentrates, described in previous work^17^, were used to perform new quantitative microprobe analyses to quantify their chemical composition and especially Ir and Ru. Primary (unmodified magmatic) phases include predominant laurite and subordinate erlichmanite (both in solid solutions). The laurite crystals are euhedral to subhedral indicating a melt-derived origin. The atoms of Ru per formula unit (apfu) in the laurite range from 0.42 to 0.73 and average 0.59. The PGM contain variable amounts of osmium (0.16–0.43 apfu) with a mean of 0.27 apfu.

**Figure 2:**
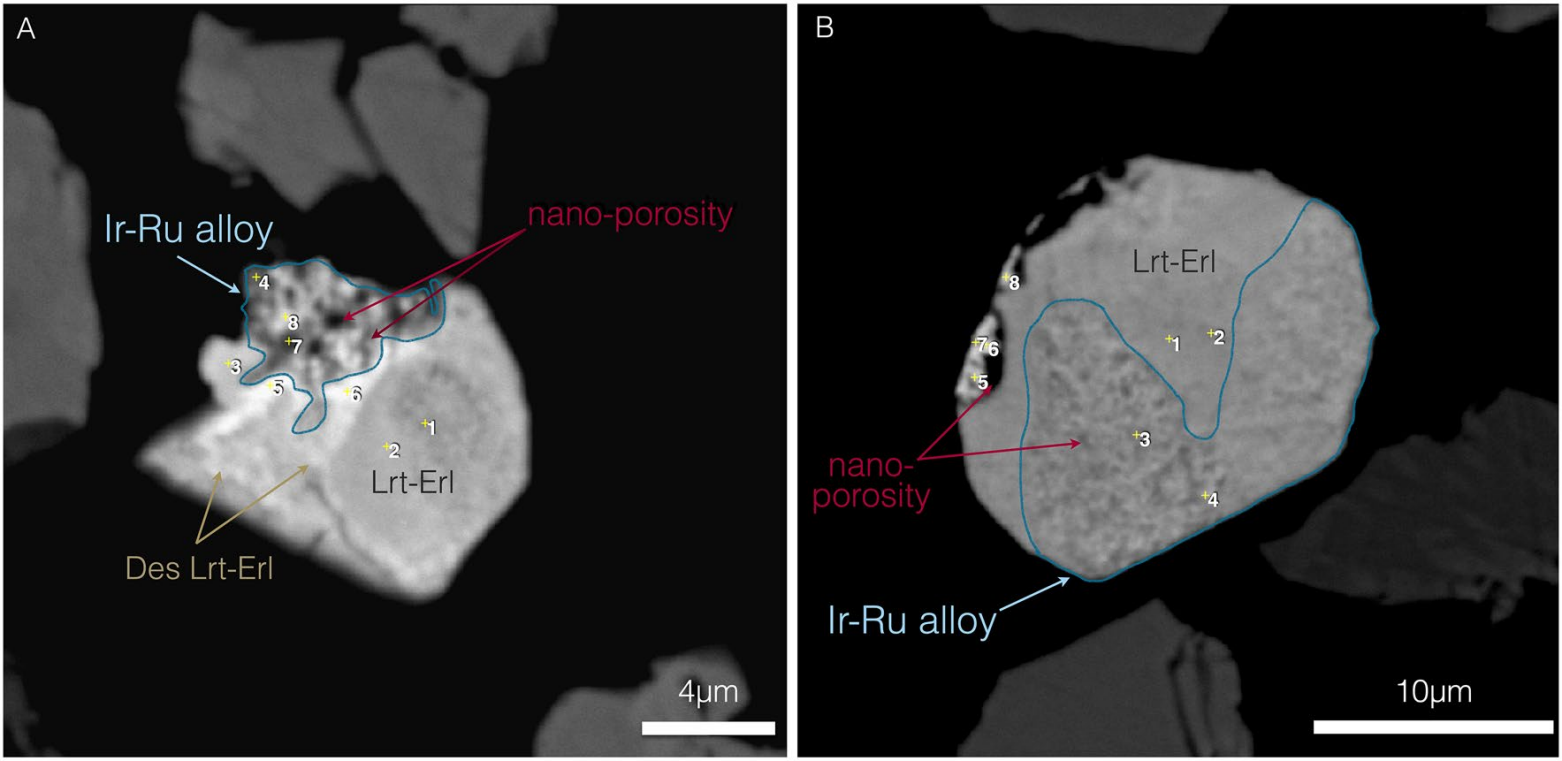
Potential catalytic sites on Ir–Ru alloys with porous structure. (**A**) anhedral, porous structure of the Ir–Ru alloys, limited to one side of the original PGM crystal. The scale bar is 4 μm, (**B**) extensive replacement of the original PGM by Ir–Ru alloys. The scale bar is 10 μm. Abbreviations; PGM: platinum group minerals. Lrt: laurite, Erl: erlichmanite, Des: desulphurised.

Iridium-bearing PGM alloys and/or sulpharsenides incorporate trace amounts of nickel and have a secondary origin (resulting from the chemical modification/alteration of primary minerals) which is evident by their textural features. They include micrometric, subhedral to anhedral Ir–Ru–Os–Ni alloys, irarsite-osarsite [(Ir,Os)AsS] solid solutions and desulphurised laurite. These secondary phases replace laurite and erlichmanite crystals propagating inwards from the outer part of the crystal. This process results in composite crystals, where the primary minerals have a uniform, solid texture, and the secondary ones show a typical spongy texture related to mass loss during alteration (Fig. 2; see also Fig. 5^17^). The compositions of the Ir–Ru–Os–Ni alloys and irarsite-osarsite are highly variable and controlled by the original composition of the primary PGM, the alteration rate, and local factors, such as the secondary micro-porosity.

### Altered PGM and H_2_ flow

The progressive removal of sulphur during laurite alteration (likely escaping as H_2_S) resulted in the stoichiometrically S-poor, variably desulphurised laurite. Mobilisation of ruthenium and sulphur from the primary laurite-erlichmanite solid solutions left a richer-in-osmium laurite relic. Desulphurisation of laurite is associated with reducing and low *f*S_2_ conditions^18–20^, indicating log *f*S_2_ below approximately − 20.5. Serpentinisation is a well-known, highly reducing alteration process. The hydration of ultrabasic rocks produces high amounts of H_2_, which lowers the *f*O_2_ and *f*S_2_ of the rock system^21–23^. Under such conditions, the PGE (Ru, Ir, Os) and base metals (e.g., Ni) are remobilised and form secondary alloys and S-poor, Ni-rich sulphides. The ultra-low *f*S_2_, coupled with a low *f*O_2_ in the H_2_-rich serpentinising fluids, is consistent with the formation of the secondary awaruite in the chromitites, which indicates log *f*O_2_ < − 35 and low water/rock ratios^23–25^. The latter implies that the reducing agent (H_2_), affecting the chromitites, was most likely in the gas state. Several secondary Ni–Co–V phosphides in the chromitites indicate ultra-high reducing conditions in these rocks^26–31^. Their secondary origin explains the statistical correlation between cobalt, vanadium, and methane in the present results.

### Rerouting the mineral-catalyst target

The multi-approach data analysis indicates the occurrence of a Sabatier catalyst, consisting primarily of iridium and ruthenium, within chromitites. Laurite (RuS_2_) is the most abundant ruthenium-bearing PGM and can incorporate up to 16 wt.% iridium. However, there is no dependence between sulphur and methane. Thus, it is highly unlikely that laurite is the catalyst in the samples. The laurite abundance cannot explain why some chromitites have considerable methane (e.g., CH_4_: 8500 ppmv, Ru: 101 ppb) and others do not (e.g., CH_4_: 1379 ppmv, Ru: 150 ppb). The current study shows that both iridium and ruthenium are critical Sabatier proxies. However, there is no linear relationship between their abundance and methane concentration. Hence, either different minerals catalyse the Sabatier reaction within different samples or a mineral with a highly varying composition is the catalyst. There is insufficient evidence to reject either possibility. However, observations indicate that an iridium-ruthenium-bearing mineral with a highly varying composition may have the strongest impact. It is possible that the secondary Ir–Ru–Os–Ni alloys are the main (but not necessarily the exclusive) Sabatier catalysts. These alloys are extremely inhomogeneous and show unsystematic metal ratios^17^, indicating that their compositions are highly influenced by the composition of the precursor laurite and erlichmanite, the variable intensity of alteration, and mobility of S, Ni and As. This variability explains their non-linear relationship with methane abundance. Moreover, their nano- spongy texture increases the available specific surface area for reactant adsorption, rendering them the ideal loci for a Sabatier reaction. There are few studies which have examined synthetic catalysts with the composition of laurite for deactivation resistance^32^. However, most of the research is focused on metallic, hybrid or metal–organic framework composite catalysts for higher efficiency^33,34^. The Ir–Ru–Os–Ni alloys are the closest natural counterpart to a metal catalyst. Secondary PGM, like the Ir–Ru–Os–Ni alloys, occur either as inclusions in spinels, or in micro-fractures filled with other secondary minerals. Previous work on the same PGM concentrates^17^ showed that the secondary Ir–Ru–Os–Ni alloys were preferentially liberated in the same fraction as magnesiochromite and not in the secondary minerals fraction. Thus, the Ir–Ru–Os–Ni alloys were inclusions in magnesiochromite, deriving from the *in-situ* destruction of laurite.

However, all catalysts require a support which is of great importance. One of the preferred supports in catalytic experiments, alumina (Al_2_O_3_), is almost identical to natural spinels (compositionally and crystallographically). Considering the Ir–Ru–Os–Ni-alloys as the catalysts, then the catalyst support is laurite. The Ir–Ru–Os–Ni-alloys-laurite composite grains are in turn supported by the magnesiochromite crystals. Hence, the noble metal alloy (hosted in laurite) inclusions in spinels are the closest natural counterpart to a metal catalyst on an alumina support.

## Discussion

A multi-discipline methodology is developed to discern the effect of the chemical composition and methane formation in a natural, multivariate chemical system of rock samples. Stochastic machine-learning algorithms, chemical analyses and microscopic observations are used to validate the current inferences. Machine learning showed that iridium concentration is the most important predictor of methane concentration in chromium-rich rocks. Ruthenium is a useful proxy for methane formation when considered in tandem with iridium. This unexpected result allowed for a shift in focus from laurite (RuS_2_)^10^ to the secondary Ir–Ru–Os–Ni alloys, as the potential Sabatier catalysts.

**Figure 3:**
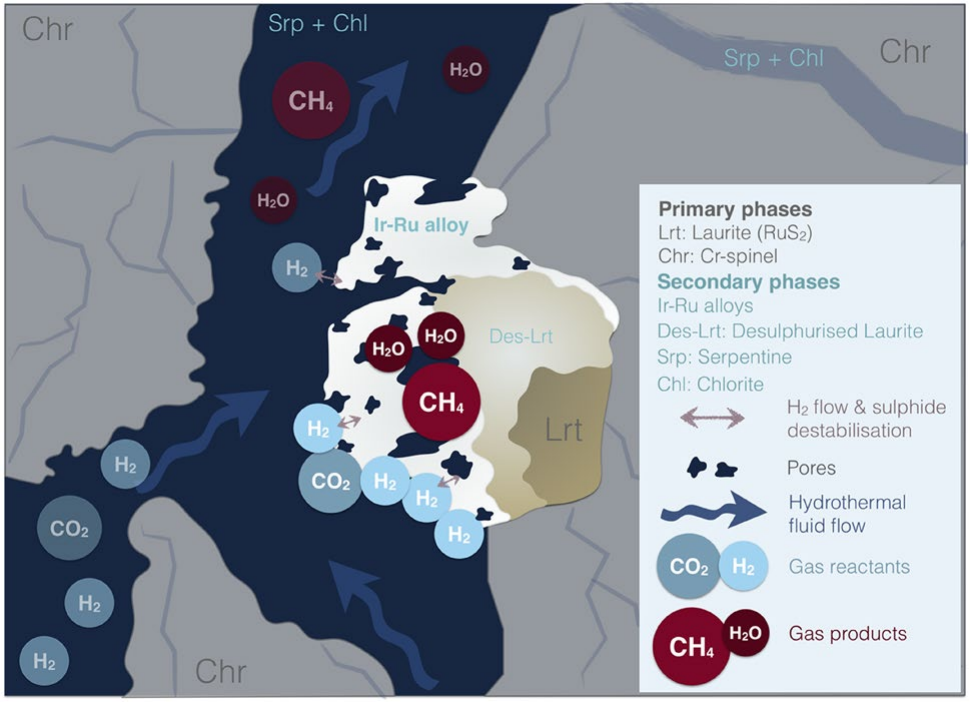
Suggested mineral catalyst. Model of the potential catalytic methanation sites of Ir–Ru–Os–Ni alloys.

Laurite is the most widespread noble-metal-bearing mineral in chromium-rich rocks, therefore its consideration as the catalyst fails to explain why methane concentrations are lower than expected (e.g., < 3000 ppmv) in many ruthenium-rich chromitites (Ru > 100 ppb), Sulphur is a common poison to the activity of a catalyst, and thus the secondary Ir–Ru–Os–Ni alloys represent a more promising catalyst target. These alloys are formed from the extreme desulphurisation of laurite that is causing mass loss and subsequently creating a nano-porous crystal surface. The Ir–Ru–Os–Ni alloys are the ideal loci of low-temperature CO_2_ hydrogenation (Fig. 3) due to their large specific area and pure metal form. Continuous flow of H_2_ gas generates extreme reducing conditions and triggers desulphurisation and formation of these alloys, in a process which may be an analogue of the routine pre-treatment methods used in catalysis to activate the metal catalysts and remove any adsorbed contaminants. The Ir–Ru–Os–Ni alloys are two-tiered supported catalysts: laurite is the integrated, first-level support, while spinel comprises the second-level support. As PGM precipitate from magmatic fluids, the bonding between the mineral catalyst and its support is superior to any synthetic counterpart. The current data cannot verify whether iridium is the catalyst or works as a promoter to ruthenium in the Ir–Ru–Os–Ni alloys (abbreviated as Ir–Ru alloys in the image) inside chromitites. Nonetheless, the overall composition of the catalyst and the support materials (e.g., Ru, Cr, MgO) may have an undetermined synergistic effect in natural systems. (abbreviated as Ir–Ru alloys in the image) inside chromitites.

The identification of a naturally occurring mineral catalyst for low-temperature CO_2_ hydrogenation is critical for sustainable catalysis. The comparatively reduced processing required for mineral catalysts would greatly reduce the carbon imprint, and the cost of the end-product. The closest synthetic counterpart catalyst is metal ruthenium with an iridium promoter on an alumina support. Pure ruthenium and iridium commonly derive from the extensive processing of chromitites. Alumina is a widely used catalyst support. However, alumina derives from energy-intensive processing of bauxites (aluminium-rich rocks). Herein, a naturally occurring, noble metal catalyst is identified with in chromitites, having an integrated support (laurite and spinel), thus making such natural chromitites an excellent, low-cost material for direct catalytic hydrogenation.

Recent studies highlight that traces of ruthenium can be extremely active catalysts^35^. Therefore, the small amounts of iridium and ruthenium detected in the studied rocks may be considered as a positive factor^10,35^. Nonetheless, the studied chromitites have evidently catalysed the low-temperature hydrogenation in the past^7,8^. It is critical to note that noble and precious metals, used for catalyst production, are already under risk for future supply disruption^36^. Thus, the use of natural catalytic materials will greatly benefit the efforts for sustainable catalysis. Minimally processed natural materials require less energy and have an immense contribution to the decrease of waste. Mineral catalysts such as the noble metal alloys in chromitites might be further investigated for their potential industrial applications.

## Conclusions

This study highlights the role of natural noble metal alloys as hydrogenation catalysts. Stochastic machine learning on whole-rock chemical data revealed that iridium followed by ruthenium are proxies of the mineral catalyst. Iridium and ruthenium concentrations were the top predictors in identifying rocks with high levels of methane and may be critical for future material exploration. In this study, the richer-in-methane, refractory chromitites host 17–45 ppb of iridium, and 73–178 ppb of ruthenium.

Microscopic characterisation and mineral analyses showed that nano-porous Ir–Ru–Os–Ni alloys comprise the catalyst. These noble-metal alloys replace *in-*situ laurite, which occurs as inclusion within fractured spinels. Laurite and spinels constitute a two-tiered support for the mineral catalyst. The noble metal alloys, laurite and spinels are naturally fused during magmatic and post-magmatic processes. The natural fusion creates bonding between the catalyst and the support, far superior to their common synthetic counterparts.

Noble-metal alloys found in chromitites can potentially serve as low-cost, sustainable catalysts for green energy production. Efficient application of mineral catalysts will drastically improve the economic viability of sustainable synthetic fuel production and have a positive environmental impact (by contributing to carbon sequestration). Additionally, natural catalysts would reduce the energy-consumption impact because of (a) the lower energy requirements for reduced processing and (b) reaction temperatures. Additional experimental work is required to determine the commercial suitability of natural noble metal catalysts within chromitites.

## Methods

### Material characterisation

A total of 12 chromitites, 11 ultrabasic rocks, 4 basic rocks, 5 rodingitised basic rocks, and 26 basic volcanic rocks comprise the dataset. All samples were collected in ophiolitic and volcanic rock outcrops in the central and northern parts of Greece. Chromitites were collected from abandoned mining sites in the following areas: Moschokarya, Eretreia, Aetorraches and Skoumtsa.

Comprehensive description on the sampling locations and detailed macroscopic, microscopic and chemical characterisation of the samples is available in the unpublished dataset of the first author^37^. The separation and extraction process of the restudied PGM concentrates and methane extraction methods and values are given in earlier works^7,17^. Additional information may be requested by contacting the first author. The whole-rock chemical analyses determined the concentrations of 55 elements and CH_4_. The analysed elements/oxides are Au, Ba, CaO, Ce, Co, Cr, Cs, Cu, Dy, Er, Eu, Fe_2_O_3(T)_, Ga, Gd, Ge, Hf, Ho, Ir, K_2_O, La, Lu, MgO, MnO, Na_2_O, Nb, Ni, Os, P_2_O_5_, Pd, Pr, Pt, Rb, Rh, Ru, S, Sb, Sc, SiO_2_, Sm, Sr, Ta, Tb, Th, TiO_2_, Tm, U, V, W, Y, Yb, Zn, Zr.

### Statistical analysis

We used centred-log ratio (clr) transformed geochemical data to perform statistical analysis in 58 samples. Values below the detection limit are replaced by ½ of the limit, while missing values are imputed using the median value representing each rock type. Data transformation is necessary as geochemical data are compositional and are prone to spurious correlations^38^. CoDa Pack app^39^ was used to perform the clr transformations (Supplementary Table 12). ANOVA was performed to detect differences between the rock types and the methane concentration. Correlations between methane concentration and the whole rock composition were performed to identify which elements have significant positive correlations with methane as candidates for catalyst proxy. Despite the data transformation, several elements continue to show outliers. Hence the non-parametric correlations (Spearman's r) on the transformed data were preferred to avoid biases. Nevertheless, Pearson's r was calculated for comparison purposes and showed almost identical results. While clr transformation has several advantages (e.g., the data are plotted in the Euclidean space and the more intuitive interpretation of the results), it does not open up the data for variables to vary independently; hence, collinearity issues are not solved. Regression is a necessary step in identifying whether the positively correlated elements show an effect on the amount of methane measured. However, Post-hoc tests on linear regression efforts revealed collinearity issues and model bias. Thus, we employed a combination of Machine-learning techniques on the untransformed data to overcome such issues and study our dataset.

### Random forest (RF) regression

The RF regression algorithm^40^ is a supervised machine-learning algorithm that uses ensembles of regression trees built for prediction, using a random number of features. The resampled data are organised hierarchically from the root to the leaf of the tree, in order to reduce variance from averaging and correlation between quantities. Random Forest is chosen because it is robust to missing and imbalanced data and can capture complex relations^41^. Furthermore, it is not multivariate-collinearity-sensitive and can handle a large number of features (see also Supplementary Notes 1 and 2).

**Figure 4:**
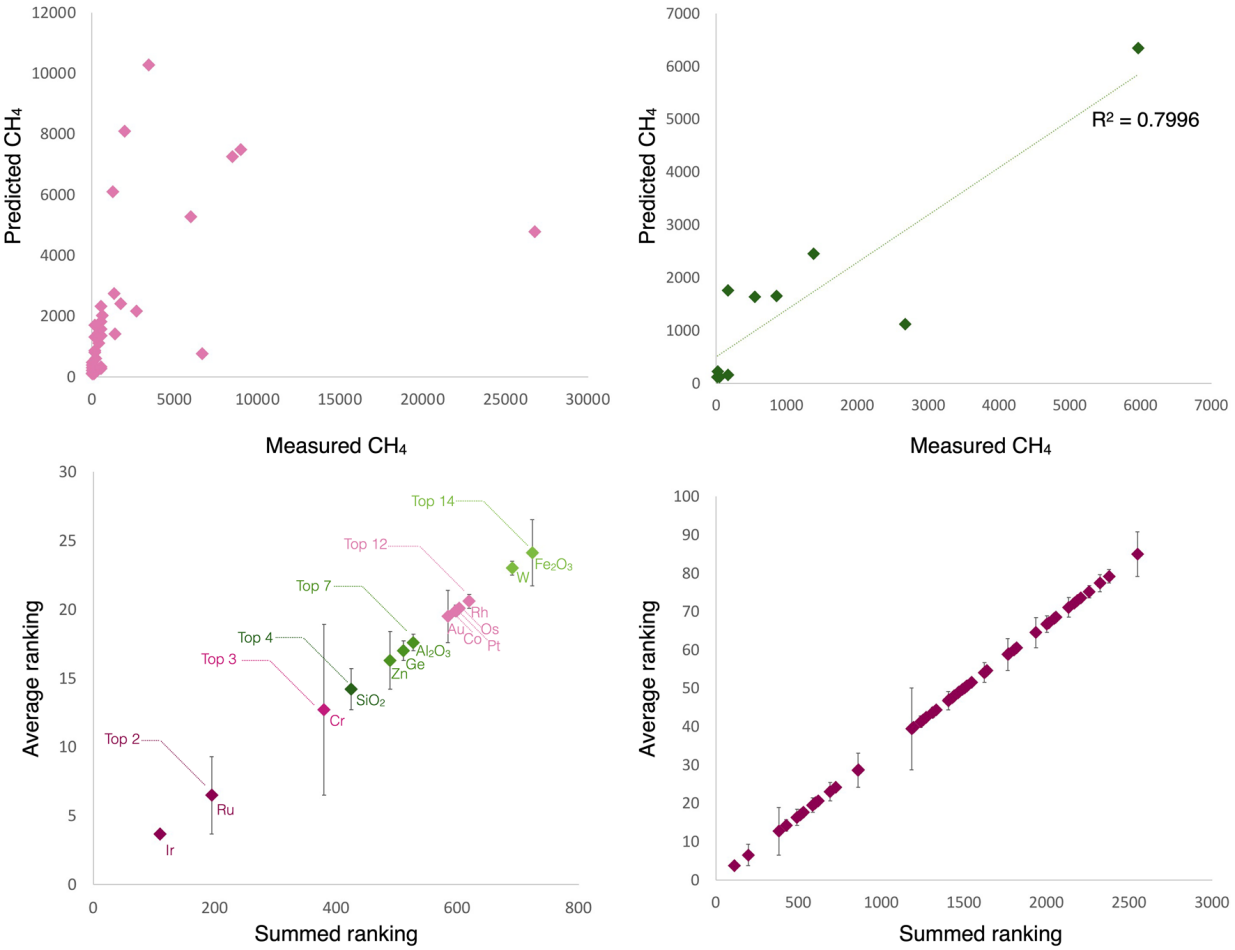
Random Forest Regression results**.** (**A**) Predicted and actual CH_4_ values during training, (**B**) Predicted and actual CH_4_ values during testing, (**C**) Top 14 features ranking, (**D**) All features ranking.

To identify the important features that contributed to the predictive performance of methane, we developed and evaluated the RF regression model as follows. We implemented this experiment using the *caret* R library^42^. The RFR experiment is repeated 30 times. For each run, we extracted the ranks of the variables using the *varImp* function and summed them over the runs. We extracted the top 20 features based on the summed ranks of features across the 30 runs. The reported train R^2^ is obtained from the getTrainPerf function from R's caret package and test R^2^ from the postResample function. The data is split into 80% train set and 20% test set. A tenfold cross-validation (CV) technique repeated over 300 times was applied on the train set. We have tried with random, leave-one-out CV, 3 and fivefold CV, over 100, 200, 400 times, and such settings gave less favourable results. We tuned the parameters using the validation set from CV, exploring ntree of 500 to 2000; and mtry of 1 to 20. The best-tuned parameters found were using 600 trees in the forest (*ntree*) and 1 variable at each node (*mtry*), studying both train and test performance metrics. Using the sum of the rank of important features generated using the *varimp* function on the 30 models, we obtained the final ranking of the 14 most important features. The last step ensured that within these 30 runs, the same important features were consistently identified. We discriminated the top feature subgroups by plotting the sum ranking against the average position and used the change in the average rank position as the error bar. According to the natural breaks observed in the plots we recognised the following top features (Fig. 4): Top 2 (Ir + Ru), Top 3 (Top 2 + Cr), Top 4 (Top 3 + SiO_2_), Top 7 (Top 4 + Zn, Ge, Al_2_O_3_), Top 12 features (Top 7 + Au, Co, Pt, Os, Rh), Top 14 features (Top 12 + W, Fe_2_O_3_). While there's a separation between iridium and ruthenium, t-SNE plotting of only two variables (Ir + CH_4_) would not be meaningful.

### t-SNE visualisation of the important features subgroups

We employed t-SNE (t-distributed Stochastic Neighbour Embedding) plots as an additional visual evaluation tool. We used these plots to cross-validate whether the identified important features for methane prediction contribute to the clustering, where each cluster reflects the methane concentration class.

The t-distributed Stochastic Neighbour Embedding (t-SNE) is a multivariate dimension reduction algorithm^16^. It represents similarity in probability distribution such that similar objects are given a higher probability value. Hence, it can reveal hidden structures in data at many different scales. The ability of t-SNE to reveal minor data structures prompted its use, as distance-based methods, such as PCA, are most efficient in displaying the variation among the methane-bearing chromitites. Due to the nature of t-SNE to express the probability distribution of similar objects as proximity, not using distance-based approaches can preserve both the local and global structure. Those of higher magnitude do not overshadow the similarity expressed here between samples with smaller ranges. Furthermore, t-SNE can handle non-linear relationships, which reflect the majority of the data deriving from natural samples, in contrast to the PCA. Additionally, the clustering of the data allows for an intuitive interpretation of the plot, contrary to a PCA.

The perplexity parameter allows the user to control the number of neighbours. We use the *Rtsne* R package^43^ on unscaled data. We set the max_iter to 6000 where the t-SNE plot has converged. Due to the stochastic nature of this algorithm, we explored different perplexity values from 2 to 19 to identify the plot with the most robust grouping (Supplementary Fig. 1). We repeated the t-SNE plotting 5 times to ensure the repeatability of the plot with the selected perplexity.

#### mBIC clustering and association measures

Model-based clustering with Bayesian Information Criterion (mBIC) creates clusters of different Gaussian-mixture models in terms of shape, volume and density using an Expected-Maximisation (EM) algorithm initialised by Hierarchical Clustering. It then uses BIC to evaluate the goodness of the clusters identified by these models. The *mclust* R package^44^ provides the *mclustBIC* and *Mclust* to create clusters and find the one with the best BIC score. Model-based clustering was chosen in this work because it is a non-distance-based clustering algorithm. We tried distance-based techniques such as k-means and hierarchical clustering, but the clusters produced were not meaningful. With mBIC, cluster memberships generated are based on probability distributions. We found that a distribution-based approach is more suitable for this dataset. The number of components (groups) was set to 5, as they are the same number of CH_4_ levels as shown in Table 1. The best model found was the EEV (ellipsoidal, equal volume and shape) model, with a BIC score of − 726.96. The association of these clusters with CH_4_ levels and lithotype are measured using the *assocstats* function from the *vcd* R package^45^ and presented in Supplementary Table 10. When using the Top 3 features, mBIC was not able to find 5 clusters and generated 4 clusters instead.

### Microprobe analyses

We performed microprobe analyses on PGM concentrate fractions for mineral characterisation and classification (Supplementary Table 12). Microprobe analyses of the PGM were conducted in the Department of Earth and Planetary Sciences, McGill University, Canada, with a JXA JEOL–8900L electron microprobe analyser operated in WDS mode. Operating conditions include acceleration voltage of 15 kV and a beam current of 20 nA, with a beam diameter of about 5 mm and a total counting time of 20 s. The ZAF correction software was used, and natural and synthetic international standards were used for calibrations.

## Supplementary Information

Supplementary Tables.

Supplementary Information 2.

## Data Availability

The dataset and additional explanatory notes are available in the Supplementary files.

## Code availability

The R code used for performing random forest, t-SNE and mBIC is available at https://github.com/dtclai/sabatier-catalyst-detection.
