# Supplementary material for: Noble metal catalyst detection in rocks using machine-learning: The future to low-cost, green energy materials?
Source: Sci Rep. 2023 Mar 7;13:3765. doi: 10.1038/s41598-023-30822-6 (PMC9992457; doi:10.1038/s41598-023-30822-6)
Supplement: Supplementary file 2 — Supplementary Information 2. [file 41598_2023_30822_MOESM2_ESM.docx]

# Noble metal catalyst detection in rocks using machine-learning: The future to low-cost, green energy materials?

Elena Ifandi^1*^, Daphne Teck Ching Lai^2^, Stavros Kalaitzidis^3^, Muhammad Saifullah Abu Bakar^4^, Tassos Grammatikopoulos^5^, Chun-Kit Lai^6^, Basilios Tsikouras^1^

^1^Geosciences Programme, Faculty of Science, Universiti Brunei Darussalam BE1410, Gadong, Brunei Darussalam. elena.ifandi@ubd.edu.bn, basilios.tsikouras@ubd.edu.bn

^2^School of Digital Science, Universiti Brunei Darussalam BE1410, Gadong, Brunei Darussalam. daphne.lai@ubd.edu.bn

^3^Department of Geology, University of Patras, GR-26504 Rio-Patras, Greece. skalait@upatras.gr

^4^Faculty of Integrated Technologies, Universiti Brunei Darussalam, Jalan Tungku Link BE1410, Brunei. saifullah.bakar@ubd.edu.bn

^5^SGS Canada Inc., 185 Concession Street, PO 4300, Lakefield, ON K0L 2H0, Canada. Tassos.Grammatikopoulos@sgs.com

^6^Fortescue Metals Group Ltd., 87 Adelaide Terrace East Perth, WA 6004. kit.lai@fmgl.com.au

Supporting Information

**Supplementary Note 1 – Classification of Machine Learning methods**

Dimension reduction algorithms

Distance-based:

- PCA – Principal Component Analysis

Stochastic:

- t-SNE – t-distribution Stochastic Neighbour Embedding
- RFR – Random Forest Regression

Clustering algorithms

Distance-based:

- k-means clustering

Stochastic:

- mBIC – modified Bayesian Information Criterion

**Supplementary Note 2 – Comment on dimension reduction and clustering methods for compositional data**

We started from the simplest dimension reduction methods for our visual outputs. We tested the efficacy of PCA on the already reduced dataset, derived from our Random Forest Regression. We performed preliminary tests on the Top 16 (Ir, Ru, Cr, SiO_2_, Zn, Ge, Al_2_O3, Au, Co, Pt, Os, Rh, W, Fe_2_O_3_, V and Ga) features. The intrinsic emphasis of PCA on linear combinations where the first principal component will have the largest variance and the last component the smallest one, is not ideal to detect local similarities. On the contrary, this method targets to highlight the dissimilarities among the data revealing only global similarities. In addition, the linear combinations of raw, compositional data may result in biased interpretations. All attempts for scaled PCA gave worse sample grouping than the unscaled ones. Additional, preliminary PCA testing with centered-log ratio transformed (clr) values, did not improve the output comparing to raw data. Clustering with unweighted k-means plotted on PCA improved a bit the overall sample grouping but the two principal components reflect mainly other large-scale geological processes (global similarities), which are only indirectly related to methane: specifically, PC1 reflects processes related to the formation and classification of the various rock types and PC2 could be better interpreted as indication of rock alteration processes.

We want to highlight the several drawbacks of PCA and k-means clustering for raw, whole-rock geochemical data:

- Dependency on the Euclidean distances between the points and the fact that raw geochemical data do not follow such a geometry
- Scale differences (i.e., when variables with large values are compared with variables with small values) can produce artificial results
- Data scaling distorts the effect of geological processes represented by high and low variances, affecting the interpretation of the results
- Interpretation of the principal components may vary largely according to the number of features used
- Complicated interpretation of an already intricate, multivariate system

**Supplementary Figure**
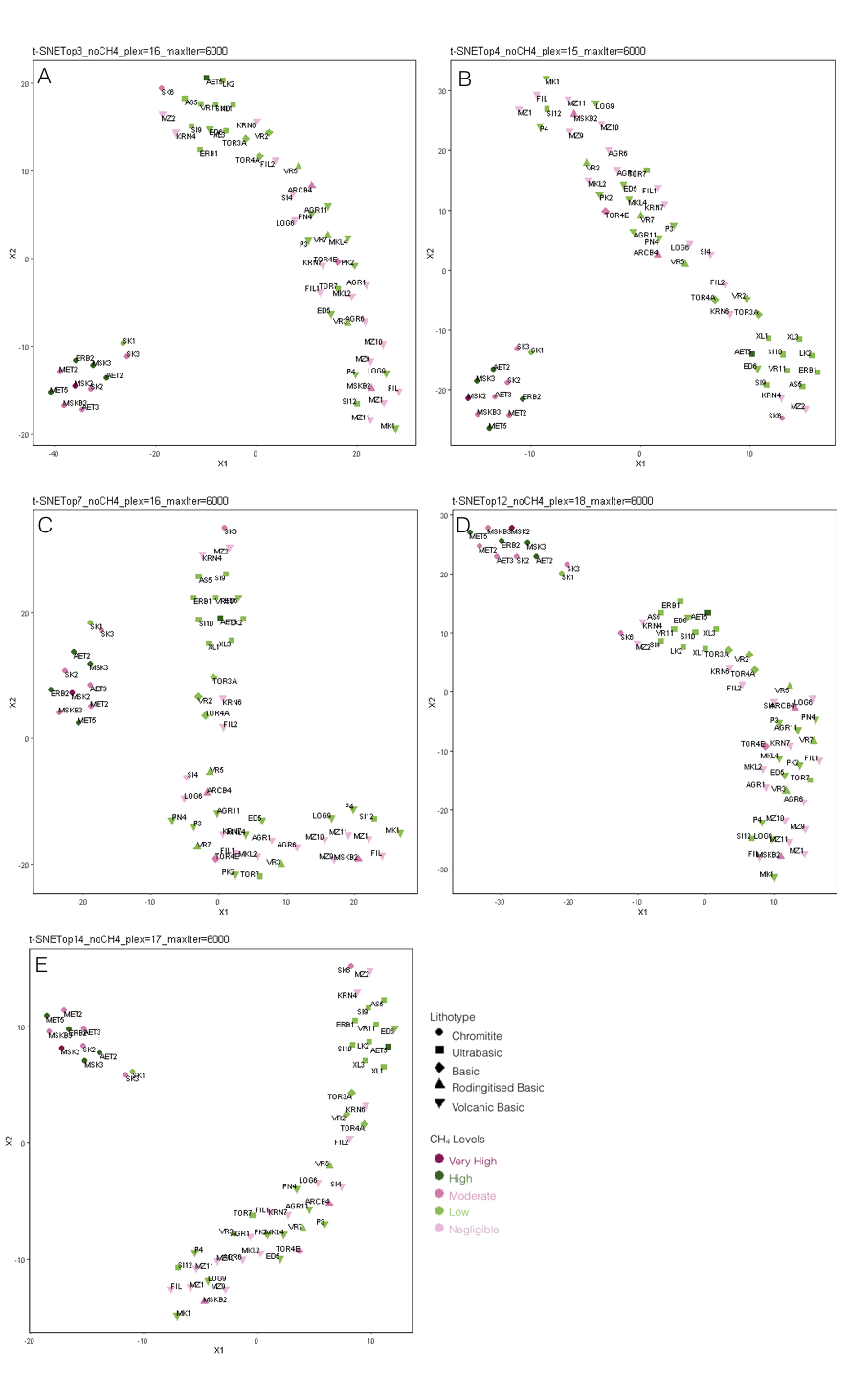


**Supplementary Figure 1.** Optimal t-SNE plots of the potential catalyst proxies at 6000 iterations. A Top 3 features (Top 2 + Cr), B Top 4 features (Top 3 + SiO_2_) C Top 7 features (Top 4 + Zn, Ge, Al_2_O_3_), D Top 12 features (Top 7 + Au, Co, Pt, Os, Rh), and E Top 14 features (Top 12 + W, Fe_2_O_3_). Abbreviations, plex: perplexity value, maxIter: maximum iterations (repetitions) of the algorithm.
